# Use of Botulinum Neurotoxin in Ophthalmology

**DOI:** 10.4274/tjo.57701

**Published:** 2016-12-01

**Authors:** Emel Başar, Ceyhun Arıcı

**Affiliations:** 1 İstanbul University Cerrahpaşa Faculty of Medicine, Department of Ophthalmology, İstanbul, Turkey

**Keywords:** Botulinum toxin, blepharospasm, hemifacial spasm, Strabismus

## Abstract

Botulinum neurotoxin (BoNT) is the first biological toxin used in the treatment of ophthalmic diseases and to decrease skin wrinkles as an aesthetic agent. When used appropriately, it weakens the force of muscular contraction and/or inhibits glandular secretion. The most common areas for botulinum toxin treatment are the upper face, including the glabella, forehead, brows, and lateral canthal lines, or crow’s feet. By relaxing the muscles causing wrinkles, non-permanent results may be achieved with its use. BoNT has gained widespread use in a variety of ophthalmic diseases. The effect of BoNT is temporary, but the therapeutic benefit is usually maintained even after repeated injections. Treatment is usually well tolerated. Complications and side effects associated with the treatment are rare and temporary. Complications occur due to weakness (chemodenervation) of adjacent muscle groups, immunological mechanisms and injection technique. Current therapeutic indications, doses, complications and contraindications of BoNT use in the following disorders related to ophthalmology were investigated: aesthetic use, strabismus, blepharospasm, hemifacial spasm, eyelid retraction, entropion, lacrimal hypersecretion syndrome, and facial paralysis.

## INTRODUCTION

Botulinum neurotoxin (BoNT), which causes the disease botulism in humans, is produced by the spore-forming, anaerobic, gram-positive bacillus bacteria Clostridium botulinum. BoNT is the most potent toxin known to humans.^1^

BoNT, the first biotoxin identified, was first applied experimentally in 1973 by Scott et al.^2^ to treat strabismus (horizontal muscles) and began to be used in humans in 1980.^3^ BoNT type A (BoNT-A) was approved by the U.S. Food and Drug Administration (FDA) in for the treatment of strabismus, blepharospasm and hemifacial spasm in 1989 and later for administration to the glabellar area for esthetic purposes in 2002.^4^ In Turkey, the Ministry of Health authorized the use of Botox (Allergan, Inc., Irvine, CA, USA) in 2001 and Dysport (Medicis Pharmaceutical Corp., Scottsdale, AZ, USA) in 2002.

It was noticed that patients treated with BoNT for blepharospasm showed a decrease in facial wrinkles, which accelerated the research and implementation of BoNT used to treat wrinkles.^5,6^ BoNT is now commonly used worldwide for esthetic purposes. Furthermore, the anhidrotic effect of the toxin was noticed after its application in neurologic diseases, and BoNT began to be used in the management of hyperhidrosis in 1994.^7^

### Mechanism of Action - Pharmacology

Clostridium botulinum is a gram-positive, anaerobic bacillus with seven antigenically unique serotypes (A-G). The neurotoxins produced by these serotypes differ in molecular size, ranging from 300 to 900 kilodalton (kDa) (Table 1). BoNT consists of a 50 kDa light chain and a 100 kDa heavy chain connected with disulfide bonds.^8^ The A, B, E, F and G serotypes cause botulism in humans.^9^ Type A is the most potent exotoxin, and is also the BoNT type most commonly used commercially. BoNT’s mechanism of action is based on block the release of acetylcholine from the presynaptic nerve terminals. In addition to acetylcholine, BoNT also inhibits the release of other chemical stimulants such as noradrenaline, dopamine, serotonin, gamma aminobutyric acid, glycine and methionine-enkephalin peptide.^8^

The diffusion of BoNT is clinically important in terms of the development of side effects to the toxin. Due to their rapid disintegration after injection, the various protein complexes used in BoNT formulations are believed to not influence the diffusion of BoNT. The administration of BoNT in large volumes or at high doses increases the area of diffusion, thereby increasing the potential for side effects.^10^

### Formulations Used in Clinical Practice

There are currently four commercial preparations of BoNT: Botox (Allergan, Inc., Irvine, CA, USA), Dysport (Medicis Pharmaceutical Corp., Scottsdale, AZ, USA), Myobloc/Neurobloc (Solstice Neurosciences, Inc., Louisville, KY, USA), and Xeomin/Bocouture (Merz Pharmaceuticals, Frankfurt, Germany) (Table 1). There are some differences between BoNT-A products. In 2009, the FDA stated that the potency of each BoNT-A product is determined by its method of preparation. In clinical practice, it is recommended to apply Botox or Dysport at a ratio of 1:2.5-3 units (U).^10,11^ This dosage was determined based on safety rather than efficacy.^12^ One U of Botox is equivalent to 1 U of Xeomin and 50-100 U of Myobloc.

One vial of Botox contains 100 U, one vial of Dysport contains 500 U, and one vial of Xeomin contains 50 or 100 U (there are two forms available) of BoNT-A. Mybloc is a BoNT-B preparation that comes in 3 different versions containing 2,500-10,000 U/vial. Unlike the other BoNT products, Mybloc requires no dilution and is ready for direct application.^10^

### Preparation and Storage Conditions

As Botox is more preferred for ophthalmic, dermatologic and cosmetic applications, the discussion of administration and dosage will focus primarily on this product.^4,6,10,11^ BoNT-A preparations are distributed in the form of vials containing lyophilized powder. A Botox vial containing 100 U of BoNT-A is reconstituted with 1-8 mL of sterile saline. The resulting 0.1 mL of Botox solution contains between 1.25 and 10 U of BoNT-A.^13^ In clinical practice, the most common dosage is 2.5 U/0.1 mL obtained by reconstituting the Botox vial with 4 mL of sterile saline. A Dysport vial containing 500 U can be reconstituted with 2.5-5 mL of saline (10-20 U/0.1 mL).^14^ BoNT is very fragile, and thus care must be taken not to foam or agitate the solution when preparing it for use. The manufacturer recommends using the BoNT solution within 4 hours or reconstitution. BoNT should not be frozen after preparation; the solution must be stored at 2-8 °C and used within 24 hours. Studies have reported that BoNT-A preparations reconstituted with isotonic sodium chloride solution and stored at 2-8 °C can be safely used for up to 2 weeks without any noticeable decrease in clinical efficacy.^11,15,16^

In addition to maintaining efficacy, preserving the preparation’s sterility is another important consideration. Alam et al.^17^ demonstrated that the sterility of a single vial of BoNT-A was not compromised by injections performed at various times over a period of 7 weeks (of a total of 127 vials, each was used an average of 4.5 times). 

### Duration of Effect

BoNT begins to take effect within 24-72 hours and reaches maximum effect within 7-14 days. Its effect on autonomic nerves (in the treatment of hyperhidrosis, overactive bladder) lasts significantly longer (6-9 months) than its effect on striated muscle (for facial wrinkles; 3-4 months).^13^

### Administration and Anesthesia

A 1 mL syringe with a 30-gauge needle is preferred for BoNT injection. Prior to injection, the skin should be cleaned with an alcohol-free antiseptic solution and dried. Amide-derivative topical creams containing a combination of lidocaine and prilocaine may be used to reduce the sensation of pain. The skin is stretched taut to reveal superficial blood vessels that should be avoided during injection.^18^

### Areas of Use

BoNT was approved by the FDA for the treatment of strabismus, blepharospasm and hemifacial spasm in 1989 and for esthetic purposes in 2002.^4^ Since its approval in 2001 by the Turkish Ministry of Health, it has become widely used in Turkey for esthetic purposes. Besides ophthalmology, BoNT is also used in various branches of medicine for pain management and functional therapy. For both men and women, the ideal age group for the use of BoNT to treat facial wrinkles formed by repeated muscle contraction is 40-60 years old. In ophthalmoplasty, BoNT is also used in deviation and oculoplastic disorders such as strabismus, blepharospasm, hemifacial spasm, upper lid retraction, entropion, lacrimal gland hypersecretion, facial paralysis.

### Facial Wrinkles

Heredity, age, environmental factors, and overaction of the facial muscles all play a role in the development of wrinkles.^19,20^ Lines that appear during movement or are unnoticeable during rest are called dynamic wrinkles, while lines with a pronounced appearance during rest are called static wrinkles.^21,22^ Carruthers and Carruthers23 noted that BoNT-A applied for cosmetic purposes was effective at lower doses when used in the middle and lower face compared to the upper face. BoNT interferes with muscle contraction and eliminates lines with no major local or systemic complications. The toxin is known to spread to an area of 2.5-3 cm around the facial injection site.^24^ Low-volume, high-concentration solutions are used to reduce the spread of BoNT in cosmetic applications.

### Forehead and Glabellar Wrinkles

The frontal muscle is responsible for wrinkles of the forehead area. When the frontal muscle pulls the muscles higher, horizontal lines appear in the skin of the forehead. The medial fibers of the frontal muscle are usually stronger, thus forming deep wrinkles. The horizontal wrinkles are marked while the frontal muscle is in maximum contraction, then intramuscular injections are done in 6 to 8 places with a dose of 10-15 U for Botox or 20-30 U for Dysport.^18^

The first cosmetic application of BoNT was to glabellar wrinkles. There are two muscles responsible for glabellar wrinkles: the procerus muscle contracts down toward the medial edge of the muscle and causes horizontal lines in the glabella, while the corrugator superciliaris muscle pulls down and in toward the medial end of the muscle, thus creating vertical lines in the area.^25^ According to the clinical findings obtained from many studies using different doses, 5 injections are done in a V pattern to the glabellar area with a dose of 20 U Botox^26,27^ or 50 U Dysport.^28,29,30^ The BoNT-A dosage for men is generally higher due to their thicker muscle mass. In a placebo-controlled, double-blind, randomized study of Botox use, male patients required an initial dose of at least 40 U to treat glabellar wrinkles.^31^

### Eyebrow Repositioning

Muscle position is determined by the balance of the frontal muscle (elevator), the orbicularis oculi, depressor supercili, corrugator supercilli and proserus (depressor).^25,32^

Intramuscular injection in the superior temporal aspect of the orbicularis oculi at 3 points with a total dose of about 10-15 U Botox or 30-40 U Dysport is recommended.^18^ A Botox injection (7-10 U) to the orbicularis oculi muscle, one of the brow depressor muscles, was reported to cause an elevation of about 1 mm in the mid-pupillary area of the brow and about 5 mm in the lateral canthal region.^33^ A three-point injection of approximately 6-10 U dose of Botox to the superior temporal orbicularis oculi muscle has been determined effective for lifting the brow.^33,34^ The injections are administered to the lateral third of the muscle and 1 cm from the bony margin of the orbit to avoid intraorbital diffusion. BoNT diffusion to surrounding tissues can result in diplopia (lateral rectus muscle), ptosis (levator palpebrae muscle) and excessive brow elevation (lateral frontal muscle).^35^

### Periorbital Wrinkles (Crow’s Feet)

Crow’s feet are wrinkles radiating outward from the lateral canthus due to the action of the orbicularis oculi while smiling.20 BoNT injection is performed 1 cm from the lateral margin of orbit in order to prevent the diffusion of BoNT to the lateral rectus muscle.^36^

Studies have determined that doses of 12 U of Botox37 or 30-36 U of Dysport^38,39^ divided into 3 injections are effective. Injecting BoNT too far above the lateral margin of orbit can cause superior eyelid ptosis, while injection too far below can result in zygomaticus muscle paralysis and lip asymmetry (lip ptosis).^40^ Excessive paralysis of the orbicularis oculi muscle can cause weakened eye closing.^24^

### Strabismus

BoNT was first applied ophthalmically in humans by Alan Scott as an alternative to strabismus surgery.^3^ His aim was to reduce the deviation by weakening the contracting antagonist muscle. BoNT is particularly suitable for complicated cases such as patients who should avoid general anesthesia, patients with paralytic strabismus or postoperative consecutive strabismus, patients with deviations less than 40 diopters, cases of active thyroid orbitopathy, patients with cyclic esotropia, and those who have undergone multiple strabismus surgeries.^41^

**Figure 1:**
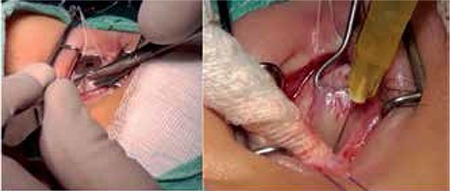
In the right eye, a fornix-based conjunctival flap is prepared from the nasal quadrant to expose the medial rectus muscle, then an intramuscular Botulinum neurotoxin injection is administered about 10 mm from the muscle insertion using a 30 gauge needle (from the Başar E. archive)

Although electromyography is usually used to facilitate the accurate injection of BoNT into the target muscle42 injection may also be done by an open method directly visualizing the muscle (Figure 1). The average dose for Botox is 1-3 U per muscle. The incidence of complications increases at higher doses (especially >10 U).^41^ BoNT-A has been reported to decrease ocular deviation in more than 50% of patients^43,44,45^ and yield satisfactory long-term results in infants and children.^46,47^

**Figure 2:**
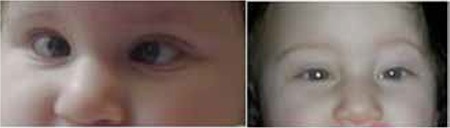
Pre- and post-botulinum neurotoxin-A injection (from the Başar E. archive)

BoNT-A injection may be used as an alternative to strabismus surgery for pediatric esotropia.^48^ Figure 2 shows a patient with infantile esotropia treated with BoNT-A (Botox) injection in our clinic. It can be seen that the patient’s esotropia resolved after Botox injection. Tengtrisorn et al.^49^ administered BoNT-A to esotropic children (mean age 26.8 months) and found that the mean angle of deviation decreased from a baseline of 40.4 prism diopters before the first injection to 24.5 prism diopters before the second injection. They reported that BoNT-A administration yielded a successful outcome in about 73% of the patients. Ruiz et al.^50^ observed success in patients older than 18 months after BoNT-A injection but reported failure in patients younger than 18 months old. In contrast, Campos et al.^51^ found that BoNT treatment was more successful in infantile esotropia patients younger than 7 months compared to patients over 7 months old. In a series of 29 cases of acute unilateral sixth nerve palsy, complete recovery of eye movements was noted in 76% of patients treated with BoNT injection to the medial rectus muscle a mean of 40 days after the onset of lateral rectus muscle palsy.^52^ In a case-control study by Yabaş et al.^53^ including 22 patients with acute sixth cranial nerve palsy, 14 patients received BoNT injection in the ipsilateral antagonist muscle and 8 were followed with occlusion therapy. Although the two groups showed comparable cure rates, the BoNT group exhibited more rapid improvement of symptoms. For chronic sixth cranial nerve palsy, transposition surgery and BoNT injection to the medial rectus muscle may be considered as a safe and effective treatment option.^54,55^

BoNT injection is also utilized as an alternative to surgery in exotropia patients. Sener and Sanac56 administered BoNT to 25 esotropia patients (mean of 1.6 injections) and 45 exotropia patients (mean of 1.6 injections) with a deviation angle of about 38 prism diopters in both groups. They reported that the angle of deviation decreased to less than 10 prism diopters in 32% of the esotropia patients and 22% of the exotropia patients. Doses of BoNT-A over 10 U were associated with increased incidence of ptosis and vertical deviation. In another study, 1.25-5 U BoNT-A administered to prevent muscle contraction in 12 sensory strabismus patients with an average deviation of 34 prism diopters provided a mean corrective effect of 73%.^57^

Residual deviation and consecutive deviation due to overcorrection are potential complications that can affect the outcomes of strabismus surgery. Various therapeutic methods can be applied in these cases, including occlusion, prismatic correction, orthoptic treatment and eyeglasses. Dawson et al.^58^ evaluated patients with consecutive esotropia following exotropia surgery and found that of 36 patients with fusion potential, BoNT-A injection resulted in an acceptable correction of deviation, resolution of diplopia and the development of high-quality stereopsis.

Approximately 80% of patients with infantile esotropia develop dissociated vertical deviation. BoNT-A injections were administered simultaneously to the medial rectus muscles of a total of 54 patients with infantile esotropia with accompanying dissociated vertical deviation divided into 2 groups by age (group 1<18 months; group 2>18 months). Complete correction of the horizontal deviation and dissociated vertical deviation was achieved in the over-18-month group.^50^

There are also reports of the benefits of BoNT administration in vertical deviations. Ozkan et al.^59^ observed that BoNT-A administered to the inferior rectus muscle in cases of adherence syndrome reduced the need for secondary surgery. BoNT injection to the inferior and superior rectus muscles was determined to effect improvement of vertical deviations in thyroid eye disease.^60^

In addition to strabismus, BoNT is also applied in nystagmus. Application is performed to multiple horizontal rectus muscles or the retrobulbar area. In some cases, retrobulbar BoNT injection causes a significant reduction in nystagmus,^44^ though unfavorable results have also been observed using this method.^61^ The dosage used in retrobulbar injection is often higher (20-30 U) than that used in intramuscular injection to the recti. Reported side effects include ptosis, diplopia, inferior rectus palsy, and total ophthalmoplegia.^41^ Carruthers^62^ applied BoNT-A to the horizontal rectus muscles of 4 congenital nystagmus patients and observed acceptable nystagmus correction and visual improvement in 3 of them. Half of the patients received repeated BoNT injections every 3-4 months to maintain their visual acuity. None of the patients developed retrobulbar hemorrhage, ptosis or globe perforation.

### Benign Essential Blepharospasm

**Figure 3:**
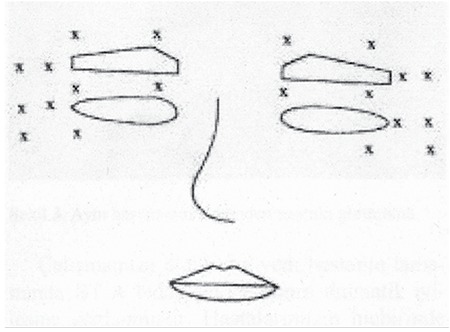
Injection spots for botulinum neurotoxin in the treatment of blepharospasm^58^

Essential blepharospasm is a focal cranial dystonia involving the eyelids and forehead muscles. It is characterized by frequent, involuntary contraction of the orbicularis oculi muscle, causing forceful closure of the eyes. Essential blepharospasm can lead to functional blindness due to involuntary eye closure. This can, in turn, have a substantial impact on patients’ personal and professional lives.^63^ Blepharospasm is more common among females.^64^ Other than greater symptom severity and frequency among women, there are no significant differences in symptoms according to gender.^65^ BoNT has been used successfully in the treatment of blepharospasm since the 1980s.^4,66,67,68,69,70,71,72,73^ BoNT is injected into the orbicularis oculi muscle immediately below the skin. The injection site is often the medial and lateral aspects of the preseptal orbicularis oculi muscle in the upper and lower eyelids in order to reduce the risk of ptosis (Figure 3). The average dose is 12.5-25 U Botox or 50-100 U Dysport for each eye.^4^ Some authors have stated that increasing the dose was necessary for repeated BoNT injections over the long term,^14,71,74^ whereas other report being able to maintain efficacy with the same dose.^67,75,76^

Local side effects may include ecchymosis, hematoma, ectropion, entropion, loss of facial sensitivity, epiphora, dry eye, lagophthalmus, photophobia, diplopia, ptosis, lip drooping, and nasal discharge. Systematic side effects of nausea, fatigue and generalized itching have been reported.^71,77,78^ Of the local side effects, diplopia most disturbs quality of life. Wutthiphan et al.^79^ reported diplopia in 1.7% of a large series of 250 cases. Ptosis is one of the most common complications. Price and O’Day80 observed ptosis in 12% of their case series.

### Hemifacial Spasm

Hemifacial spasm is the unilateral, repetitive tonic or clonic contraction of the facial muscles innervated by the facial nerve. It usually begins in the fifth to sixth decade and is unilateral. In contrast to blepharospasm, hemifacial spasm continues during sleep. It is not associated with excessive sensory stimulation. Rarely, the condition may manifest bilaterally.^4^

It is treated by 25-35 U Botox^71,81^ or 47-92 U Dysport^82,83,84^ injection. Of studies with long-term follow-up of BoNT-A injection for hemifacial spasm, Ababneh et al.^71^ reported that the mean post-injection duration of effect was 14.1 weeks after 1 year and reached 18.1 weeks after 10 years. Gill and Kraft85 determined the first 10 injections to be effective for a mean of 12.4 weeks and claimed this mean duration remained stable over the following 10 injections. Akdemir et al.^86^ noted no change in duration of effect after BoNT injection in hemifacial spasm (mean follow-up 90.3 months) and increasing duration (16.1 weeks after the first 5 injections, 18.9 weeks after the last 5 injections) in blepharospasm patients (mean follow-up 51.8 months).

### Upper Eyelid Retraction

BoNT can be used for the temporary correction of upper eyelid retraction. Temporary improvement of the palpebral fissure height has been observed with doses of 2.5-10 U Botox delivered by transconjunctival injection just above the upper tarsal border into the levator-Müller muscle complex.^87,88,89^ Salour et al.^90^ reported that a single dose of 20 U of Dysport injected transcutaneously at the central superior tarsal border into the levator aponeurosis and Müller muscle was a safe and effective treatment. Ptosis and diplopia may arise as minor complications.

### Congenital and Acquired Entropion

BoNT injection reduces the tone of the pretarsal and preseptal fibers of the orbicularis oculi muscle, thereby providing temporary correction of its inward folding. BoNT-A (Botox) is injected subcutaneously in 5 U doses to each of 3 points approximately 3-4 mm below the lower eyelid margin.^91^

### Lacrimal Gland Hypersecretion

Gustatory (taste-related) lacrimation (crocodile tears syndrome) is an autonomic synkinesia causing excessive tear production. It is often idiopathic or arises secondary to aberrant reinnervation of the lacrimal gland by efferent fibers of the seventh or ninth cranial nerves in patients with history of traumatic facial palsy. A small proportion of patients may require treatment. BoNT-A injection has been shown to be effective.^92^ A transconjunctival injection of 2.5 U BoNT-A (Botox) is applied directly to the palpebral lobe of the lacrimal gland. Duration of effect is 6 months.^93,94^

### Facial Paralysis

Instead of tarsorrhaphy or gold weight implants to protect the ocular surface in cases of facial paralysis, corneal damage may be prevented by using BoNT-A injection to the levator palpebrae superioris muscle to induce eyelid ptosis. Due to the proximity of the levator palpebrae superioris muscle to the superior rectus muscle, Naik et al.^95^ recommended using a needle half the length of the standard 25 mm needle in order to prevent hypotropia and weakened Bell reflex. Yucel and Arturk96 injected 7.5 U BoNT-A (Botox) near the midline of the orbital roof and observed a mean duration of effect of 10 weeks.

### Complications and Side Effects

When used appropriately, treatment is generally safe and well tolerated by patients. As the effects of BoNT-A generally begin to fade within 12 weeks, the duration of its side effects is limited.^97^ These self-limiting side effects, which are especially common with repeated injections and occur in about 3%, include headache, edema, bruising, mild pain related to the injection and flu-like symptoms.^98,99^ Side effects like bruising and hemorrhage can be minimized by discontinuing patients’ use of anticoagulants (aspirin, vitamin E, nonsteroid antiinflammatory drugs) two weeks prior to injection. In addition, the treated area should not be massaged for up to two hours after injection in order to accelerate the absorption of the injected BoNT and reduce its spread to surrounding tissues. Patients should be warned of these issues.^24^

Blepharoptosis may occur during treatment of glabellar lines or periorbital wrinkles. Carruthers et al.^100^ observed the condition in 5.4% of their cases. It has been recommended to increase the concentration and reduce the volume of BoNT-A injections to prevent unwanted diffusion to other muscles.4 Ptosis is one of the common complications. This arises due to diffusion or accidental injection of the toxin into the orbital septum. Ptosis occurs in an average of 13% of cases.^101^ In cases of ptosis severe enough to interfere with vision, the use of 0.5% apraclonidine ophthalmic drops to enhance Müller muscle function may be beneficial the levator muscle function returns. According to a meta-analysis of 1003 patients, the most common complication was ptosis (3.4%), followed by dry eye (2.3%), headache (1.6%) and eyebrow ptosis (0.6%).^102^ Eyelid ptosis often occurs due to impairment of the levator muscle after injection to the glabellar lines invades the orbital septum. Ptosis emerges as early as 48 hours and up to 2 weeks after injection and can last from 2 to 12 weeks. To avoid eyelid ptosis due to intraorbital diffusion, a high-concentration, low-volume BoNT injection is applied 1 cm from the edge of the orbital bone or more than 1.5 cm laterally from the lateral canthus.^103^ Diplopia is a rare complication which usually occurs due to paralysis of the inferior oblique muscle. Dry eye and epiphora are other common complications of BoNT administration. Blurred vision resulting from corneal exposure may occur due to disruption of the eye-closure reflex. There have been rare reports of acute angle closure glaucoma^104,105^ and retinal tearing due to globe penetration^106^ associated with BoNT injection. To date, reported side effects include pain during injection, local edema, erythema, ecchymosis, alternate muscle weakness, flu-like symptoms and high cost. Between 1989 and 2003, nearly all of the serious complications related to BoNT injection reported to the FDA were a result of therapeutic applications using higher dosages (ratio of therapeutic:cosmetic purposes was 33:1). Of 253 cases with serious complications, 28 deaths were reported, none of which were related to the application of BoNT for cosmetic purposes.^107^

The proteins included in the preparations may cause antibody reaction against BoNT injections. The BoNT agent currently in use (since 1998) has a low protein load and therefore rarely induces an allergic reaction. However, an allergic reaction can occur due to the therapeutic use of high-dose BoNT. Of 1437 BoNT-related adverse events reported to the FDA, nonserious allergic rash occured in 17 cases of therapeutic use and 29 cases of cosmetic use, while serious allergic reaction/rash occured in 11 therapeutic users and 2 cosmetic users.^107^ Decreasing the dose of BoNT and increasing the intervals between injections can reduce the risk of antibody production. In regards to malpractice, there have been reports to the FDA of side effects due to toxin spreading to surrounding tissues after BoNT injection for cosmetic purposes, but no permanent serious side effects have been reported.^107^

### Contraindications

BoNT should not be used by pregnant (category C) and breastfeeding mothers (it is not known whether BoNT passes to breast milk); children under 12 years old; individuals with extreme sensitivity to any components of the preparation; or patients with coagulopathies or neuromuscular disease (myasthenia gravis, Lambert-Eaton syndrome, multiple sclerosis, etc.).^24,108^

The skin should not be cleaned with alcohol. Because they reduce the release of acetylcholine, the effect of the toxin is increased by aminoglicosides, cyclosporine, D-penicillamine, quinidine, succinylcholine, magnesium sulfate and lincosamides, whereas aminoquinolones reduce its effect by blocking its cellular uptake.^24,108^ Therefore, a detailed medical history must be taken prior to the application of BoNT.

## CONCLUSION

The average lifespan is longer than ever before, and the chemical denervation agent BoNT is remarkably effective in reducing the signs of aging around the eyes and face. In addition to its use in oculoplasty, BoNT has various uses in ophthalmology for eyelid and lacrimal system disorders. Furthermore, BoNT has taken its place in medicine as a powerful chemical alternative to strabismus surgery which, especially in pediatric esotropia and most types of paralytic strabismus, can be as effective as surgery without altering the muscular anatomy.

### Ethics

Peer-review: Externally peer-reviewed.

## Figures and Tables

**Table 1:**
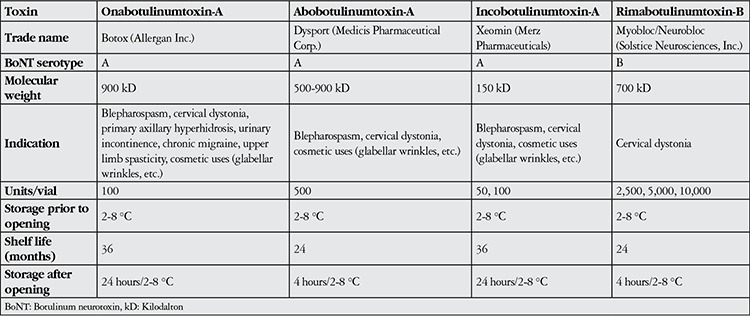
Comparison of Botulinum neurotoxin products
